# Impact of Fire and Heat Stress on Soil Microorganisms: A Review of Community Changes, Molecular Responses and Plant‐Beneficial Roles

**DOI:** 10.1111/1758-2229.70247

**Published:** 2026-03-05

**Authors:** Ma. del Carmen Orozco‐Mosqueda, Aurora Flores‐Piña, Ajay Kumar, Fannie I. Parra‐Cota, Sergio de los Santos‐Villalobos, Debasis Mitra, Olubukola Oluranti Babalola, Gustavo Santoyo

**Affiliations:** ^1^ Department of Biochemical and Environmental Engineering Tecnológico Nacional de México en Celaya Celaya Mexico; ^2^ Genomic Diversity Lab, Institute of Biological and Chemical Research Universidad Michoacana de San Nicolás de Hidalgo Morelia Mexico; ^3^ Amity Institute of Biotechnology Amity University Noida India; ^4^ Campo Experimental Norman E. Borlaug‐INIFAP Cd. Obregón Mexico; ^5^ Instituto Tecnológico de Sonora Ciudad Obregón Mexico; ^6^ Department of Microbiology Graphic Era (Deemed to Be University) Dehradun India; ^7^ Food Security and Safety Focus Area, Faculty of Natural and Agricultural Sciences North‐West University Mmabatho South Africa; ^8^ Department of Life Sciences Imperial College London Ascot UK

**Keywords:** abiotic stress, microbial diversity, soil microbiome, surface fires, underground fires

## Abstract

Fire, whether occurring on the surface or underground, significantly influences soil microbial dynamics by reshaping community composition, functional diversity and overall soil and plant health. This review examines the effects of fire on soil‐beneficial microbial communities, with particular attention to how surface and underground fires drive shifts in microbial diversity and functional roles within the agroecosystems. These changes impact key processes such as nutrient cycling, soil physicochemical structure and organic matter decomposition, ultimately affecting crop production. Bacterial groups such as *Firmicutes* and *Actinobacteria* often increase in abundance following fire events, while others lacking survival strategies tend to decline. Resilient fungal groups, including *Ascomycota* (such as *Aspergillus*, *Penicillium* and *Trichoderma*), frequently play pivotal roles during the recovery process. Fire can also enhance microbial metabolic activity, particularly in pathways involved in organic matter degradation, leading to short‐term increases in nutrient availability that support plant regrowth. Finally, the review discusses the molecular responses of microbes to fire and outlines perspectives for better understanding this type of stress and how it affects the beneficial soil microbiota in agricultural edaphic systems.

## Graphical abstract

This work reviewed the direct and indirect effects of fires on microbial communities and the resilience strategies microbes use to withstand high temperatures .

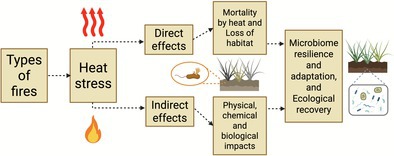

## 1 Introduction

Fires play a pivotal role in the Earth's system, and their frequency and severity are increasing due to global environmental changes. These events significantly impact soil carbon (C) and nitrogen (N)—key components of soil health and essential drivers of ecosystem services (Li et al. 2021; Roshan and Biswas 2023). While wildfires are natural phenomena, their interactions with ecosystems and the climate pose serious risks to biodiversity and human communities (Li et al. 2021; Roshan and Biswas 2023). Fire behaviour is generally classified into flaming and smouldering types, which often occur simultaneously during wildfires (Rein and Huang 2021). Underground smouldering fires can transition into surface fires, shifting from slow, oxygen‐limited burning to intense, visible flames (Zhang et al. 2024). They typically ignite organic materials such as coal deposits, tree stumps, fallen logs, large branches, duff, roots and organic soils, often as a result of spontaneous combustion, external fires or human activities (Liang et al. 2023; Rein and Huang 2021; Zhang et al. 2024).

Both surface and underground fires strongly alter the physical, chemical and biological properties of soil. Fire‐induced changes—such as soil compaction, shifts in pH and nutrient loss—directly affect soil biota and reduce the soil's capacity to support plant growth, indirectly impacting the resources available to soil organisms (Certini et al. 2021; Agbeshie et al. 2022). Microbial communities, particularly fungi of families of *Ascomycota* are known to persist in soils after forest fires due to their heat‐resistant traits. Members of Trichocomaceae, including *Aspergillus* and *Penicillium*, produce thermotolerant spores and disperse rapidly, while Hypocreaceae, such as *Trichoderma* and *Fusarium*, exhibit resilience in degraded and post‐fire soils. Chaetomiaceae (*Chaetomium*) tolerate high temperatures and contribute to the decomposition of plant material, whereas Xylariaceae (*Xylaria* and *Hypoxylon*) are capable of colonising charred wood and other burned organic matter. Other heat‐resistant microbial groups like bacteria (i.e., *Bacillus* spp.) and archaea (i.e., *Nitrososphaera*, *Nitrosopumilus*) are also spore‐forming organisms and play essential roles in nutrient cycling, organic matter decomposition and soil recovery after disturbance (Khatoon et al. 2020; Mydeen et al. 2023).

**FIGURE 1:**
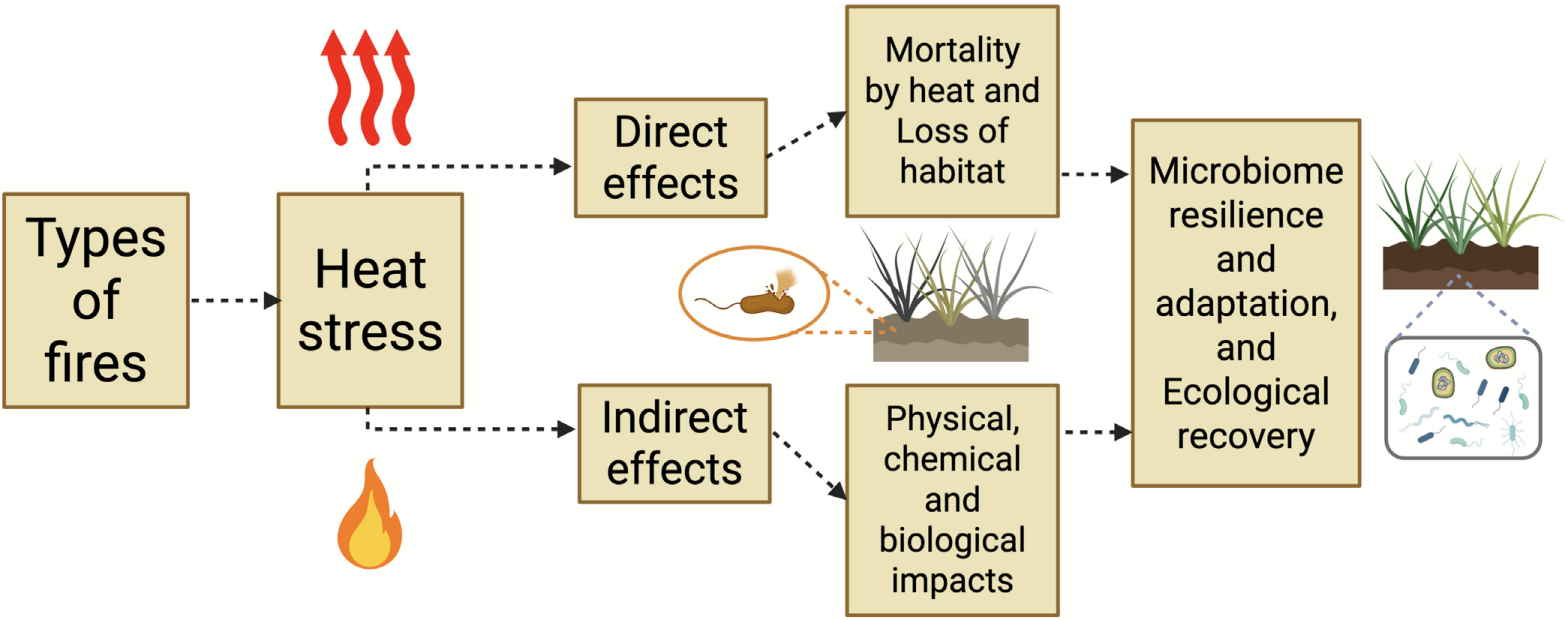
Soil microbiome responses to fires. The figure illustrates the direct and indirect effects of fires on microbial communities, with positive and negative signs indicating the impact on major soil microbial taxa. See text for further details.

Advances in molecular techniques, including high‐throughput sequencing and metagenomics, along with the increasing availability of microbial genomic data, have provided invaluable insights into the composition and dynamics of soil microbial communities under diverse environmental conditions (Fierer et al. 2017). Increasing fire activity and climate change drive shifts in microbial metabolism, with cascading effects on soil carbon and nitrogen cycling (Li et al. 2021; Hu et al. 2023; Jones et al. 2023). Understanding these microbial dynamics is crucial for developing sustainable land management strategies that preserve soil health and ecosystem functioning. This review consolidates recent findings on the structure and function of microbial communities in soils affected by surface and underground fires, highlighting how fire alters microbial diversity and ecosystem processes through both direct and indirect mechanisms (Figure 1).

## 2 Soil Microbial Communities: Diversity and Function

Soil is a complex habitat that sustains a rich diversity of microorganisms, including bacteria, fungi and archaea (Fierer et al. 2017). While some researchers consider soil a challenging environment for microbial life (Nannipieri et al. 2020), it remains a thriving habitat. A single gram of soil can contain vast quantities of microorganisms: between 10^9^ and 10^10^ prokaryotic cells (Torsvik et al. 1990; Srinivasiah et al. 2008; Fierer et al. 2017), 10^4^ to 10^7^ protists (Bates et al. 2013), up to 100 m of hyphal growth (Bardgett and Van der Putten 2014) and up to 10^10^ viruses (Kuzyakov and Mason‐Jones 2018). Advances in omic studies of soil samples have allowed scientists to unravel previously yet unknown microbial diversity (Babalola et al. 2009) and the technologies get better by the day.

Soil microbial communities are fundamental drivers of key ecosystem processes and functions, including nutrient cycling, primary production, litter decomposition, climate regulation and soil formation (Bardgett and Van der Putten 2014; Delgado‐Baquerizo et al. 2017, 2020). Among these communities, bacteria, fungi and archaea are the most abundant and ecologically dominant groups. They play critical roles in ecosystem functioning and exhibit distinct patterns of abundance and diversity across environments (Fierer et al. 2009; Fierer 2017). Consequently, microbial diversity is a valuable indicator of changes in soil processes and of ecosystem recovery following wildfire disturbances (Muñoz‐Rojas and Bárcenas‐Moreno 2019). Ecosystem recovery is often a self‐regulating process, reflecting the community's adaptive response to external pressures that alter its structure (Babalola 2014).

Fire disturbances exert complex and lasting effects on ecosystem functioning, with consequences that may persist for months or even years, depending on the responses of plant and microbial communities (Certini et al. 2005; Pérez‐Valera et al. 2020; McLauchlan et al. 2020; Certini et al. 2021). The richness (i.e., number of distinct taxa) and abundance of soil bacteria and fungi vary widely and are influenced by factors such as soil type, land use, climate, pH and management practices (Tedersoo et al. 2014; Fierer et al. 2017; Mydeen et al. 2023; Roshan and Biswas 2023).

### 2.1 Soil Bacteria

Soil bacteria are a group of prokaryotic microorganisms with high diversity in the soil, with *Proteobacteria* being one of the most abundant and diverse bacterial phyla, often accounting for 20%–50% of the total bacterial biomass in various agricultural soils (Lozupone and Knight 2007). This phylum includes species such as *Pseudomonas fluorescens* , known for its plant growth‐promoting traits, and *Rhizobium leguminosarum* , a key nitrogen fixer in legume symbiosis. Named after the Greek god Proteus for their variability in shape and physiology, Proteobacteria contribute to critical soil processes such as nitrogen fixation and organic matter decomposition (Mukhopadhya et al. 2012). Similarly, Actinobacteria are Gram‐positive bacteria with high G + C content and wide metabolic capabilities. Notable species include *Streptomyces coelicolor* , a prolific producer of antibiotics, and *Micromonospora* spp., which enhance nutrient cycling in soil. They are vital for decomposing organic materials, particularly in nutrient‐poor environments, and can constitute 10%–30% of soil bacterial communities (Lauber et al. 2009; Olanrewaju and Babalola 2019). Firmicutes are a phylum of bacteria characterised by a low G + C content in their DNA and include Gram‐positive endospore‐forming species as well as lactic acid bacteria. Prominent members such as *Bacillus subtilis* , known for its biocontrol and plant growth‐promoting properties, and *Clostridium pasteurianum* , a nitrogen‐fixing anaerobe, exhibit remarkable resistance to extreme environmental conditions due to their ability to form endospores (Filippidou et al. 2016). Although less abundant, Acidobacteria are increasingly recognised for their ecological importance in soil. Key species include *Acidobacterium capsulatum* , tolerant to low pH environments, and *Solibacter usitatus*, thriving in nutrient‐depleted soils, both contributing to organic matter decomposition and nutrient cycling (Sikorski et al. 2022). Bacteroidota also plays essential roles as decomposers, breaking down complex organic matter and enhancing nutrient availability. Representative species such as *Flavobacterium johnsoniae* , involved in cellulose degradation, and *Chitinophaga pinensis* , a specialist in chitin decomposition, are found across a wide range of ecosystems, including deserts and glaciers (Pan et al. 2023). Finally, Cyanobacteria excel in arid and semi‐arid soils due to their resilience to environmental stresses. Notable examples include *Nostoc commune* , important for nitrogen fixation and soil stabilisation, and *Microcoleus vaginatus* , a pioneer in biological crust formation in desert soils. These microorganisms enhance soil fertility and contribute significantly to soil health and agricultural productivity in nitrogen‐limited regions (Etesami et al. 2023; Büdel et al. 2016; Ramakrishnan et al. 2023).

### 2.2 Soil Fungi

Fungi are critical soil microorganisms, essential for organic matter decomposition and plant symbiosis, with soil fungal diversity reaching thousands of species within a single sample. (Hawksworth 1991). Among these, mycorrhizal fungi are particularly vital, aiding nutrient acquisition, stress adaptation and ecosystem sustainability (Igiehon and Babalola 2021; Shi et al. 2023). The largest fungal phylum, Ascomycota, includes diverse species such as *Aspergillus niger*, renowned for its enzymatic capabilities in organic matter decomposition, and *Penicillium chrysogenum*, a key producer of antibiotics. These fungi dominate soil ecosystems globally and play essential roles in promoting soil health and agricultural productivity (Egidi et al. 2019). Basidiomycota, recognised for their fruiting bodies (mushrooms), are significant decomposers of lignin and cellulose, with representative species including *Ganoderma lucidum*, a wood‐decaying fungus with medicinal properties, and *Laccaria bicolor*, a mycorrhizal symbiont that enhances plant nutrient uptake. They account for 20%–30% of fungal communities in many soils (Zmitrovich et al. 2023). Glomeromycota forms arbuscular mycorrhizal (AM) associations with plant roots, with species such as *Rhizophagus irregularis* essential for enhancing phosphorus uptake and improving soil structure (Stürmer and Kemmelmeier 2021). Lastly, Mucoromycota includes saprophytic species like *Mucor circinelloides*, which contribute to organic matter decomposition, and *Mortierella elongata*, a root‐associated fungus involved in soil nutrient cycling and plant growth promotion (Bonfante and Venice 2020).

The populations of *Trichoderma* spp. are mainly known as biocontrol agents against plant diseases, as well as promoters of plant growth, being a fundamental component in agricultural soils by maintaining the good health and productivity of crops (Guzmán‐Guzmán et al. 2023, 2024). In a recent study, a different role was found for this beneficial fungal genus: the fungus *Trichoderma harzianum* was evaluated for its ability to accelerate the degradation of flammable needles from *Pinus koraiensis* to reduce the risk of forest fires. Through 270 laboratory experiments, it was demonstrated that a dose of 4 mL of *T. harzianum* applied over 42 days was the most effective in decreasing flammability by 203%, reducing flame intensity by 54%, and shortening the flame length by 31%. Additionally, this microbial degradation significantly altered the physicochemical properties of the combustible material, limiting fire spread (Li et al. 2024). The results highlight the potential of *Trichoderma* as a biological tool for managing flammable organic matter, emphasising its role as a fire risk mitigator in forest ecosystems.

### 2.3 Archaeal Soil Communities

Archaea, though less studied than bacteria and fungi, play a significant role in soil health and stability, particularly under extreme or anaerobic conditions, by cycling key nutrients such as carbon, nitrogen, sulphur and minerals (Naitam and Kaushik 2021; Starke et al. 2021). Their resilience to harsh environments—characterised by high salinity, extreme temperature and fluctuating pH—makes them crucial for maintaining microbial diversity in challenging ecosystems (Naitam and Kaushik 2021; Starke et al. 2021). The main archaeal taxa found in soils include methanogens, ammonia‐oxidising archaea (AOA) and halophiles, each contributing uniquely to soil processes (Bräuer et al. 2020; Huang et al. 2021). Methanogens thrive in anaerobic environments such as wetlands, waterlogged soils and rice paddies, metabolising organic matter to produce methane via methanogenesis; notable species include *Methanobacterium formicicum* and *Methanosarcina barkeri* , with their activity linked to greenhouse gas emissions and global climate dynamics (Bräuer et al. 2020). Ammonia‐oxidising archaea, integral to nitrogen cycling by oxidising ammonia to nitrite, dominate acidic, nutrient‐poor or low‐ammonia soils, often outcompeting ammonia‐oxidising bacteria; key species include *Nitrososphaera viennensis* and *Candidatus Nitrosocosmicus franklandus*, with AOA comprising 10%–30% of microbial biomass in some soils (Huang et al. 2021). Halophilic archaea, typically inhabiting saline and saline‐alkaline soils, contribute to nutrient cycling and soil resilience under harsh conditions; representative species include *Halobacterium salinarum* and *Haloferax volcanii* , exemplifying remarkable adaptability to extreme environments despite being less abundant in typical soils (Martínez‐Espinosa 2024).

Because archaea are one of the groups that cannot be easily isolated and cultured in the laboratory—especially when compared to other prokaryotes—sequencing techniques targeting 16S ribosomal genes have made it possible to explore their diversity, relative abundance and functional roles in agricultural soil systems (Odelade and Babalola 2019). In a recent review, Alori et al. (2020) discussed the roles of various archaeal groups and their potential as plant growth promoters in arid and semi‐arid regions. Archaeal phyla such as *Crenarchaeota* and *Euryarchaeota* have been associated with maize crops, while *Methanobacteriales*, *Methanomicrobiales*, *Methanosarcinales* and *Methanocellales* have been frequently found in rice cultivation systems. Interestingly, a study by Song et al. (2019) reported that a plant growth‐promoting archaeon, *Nitrosocosmicus oleophilus* MY3, triggered induced systemic resistance in *Arabidopsis thaliana* against *Pectobacterium carotovorum* and *Pseudomonas syringae* . Understanding these archaeal communities—and the impact that fire may have on them—enhances our knowledge of soil ecology and informs sustainable soil management, as their roles in nutrient cycling and environmental resilience are indispensable for maintaining soil ecosystem functions (Oren 2014).

### 2.4 Soil Protists

Soil protists, a diverse group of mostly single‐celled eukaryotes, play crucial roles in soil ecosystems by regulating microbial populations, nutrient cycling and organic matter decomposition. Their diversity encompasses amoebae, flagellates, ciliates and slime moulds, each occupying distinct ecological niches and contributing uniquely to soil food webs (Geisen et al. 2018). Amoebae, such as *Acanthamoeba* spp., are important bacterial predators that help control bacterial abundance and promote nutrient mineralisation. Flagellates, including species from the genera *Cercomonas* and *Bodo*, actively graze on bacteria and smaller protists, influencing microbial community structure and dynamics. Ciliates, like *Colpoda* spp., contribute to the decomposition of organic matter and are sensitive indicators of soil health and pollution (Geisen et al. 2017). Slime moulds, though less studied in soils, participate in the breakdown of organic matter and act as biological indicators of ecosystem function. Collectively, soil protists facilitate nutrient availability for plants by accelerating microbial turnover and maintaining soil microbial diversity, thus playing an indispensable role in sustaining soil fertility and ecosystem resilience (Fiore‐Donno et al. 2020).

Recently, new evidence has highlighted the role of protists as modulators of beneficial microbial populations in the rhizosphere, and even in the endosphere, having a positive effect on plant host growth and fitness (Santoyo et al. 2025). For example, Murase and Asiloglu (2024) analysed the role of protists in wetland rice soils. In these environments fluctuating redox conditions and diffused root exudates shape microbial communities. Rice roots act as hotspots for protist attraction, influencing nutrient availability and microbial populations, where protists have been shown to modulate plant‐associated microbiota (in both paddy and non‐paddy soils), with effects ranging from beneficial to detrimental. Sapp et al. (2018) found cercozoan taxa in *Arabidopsis* rhizospheres using 18S rRNA gene metabarcoding, while other studies reported bacterivorous Amoebozoa and Alveolata in pea, wheat and oat. Asiloglu et al. (2024) further demonstrated that axenic protists— *Acanthamoeba castellanii* , *Vermamoeba vermiformis* and *Heteromita globosa*—significantly altered soil‐specific bacterial amplicon sequence variants (either enriching or depleting them), though their impact on rhizosphere and endophytic communities varied with soil type, indicating context‐dependent interactions. Protists, although not adapted to withstand high temperatures or fire conditions, play key ecological roles in soil ecosystems by regulating microbial populations and contributing to nutrient cycling.

## 3 Fire‐Driven Transformations in Soil Properties and Structure

**FIGURE 2:**
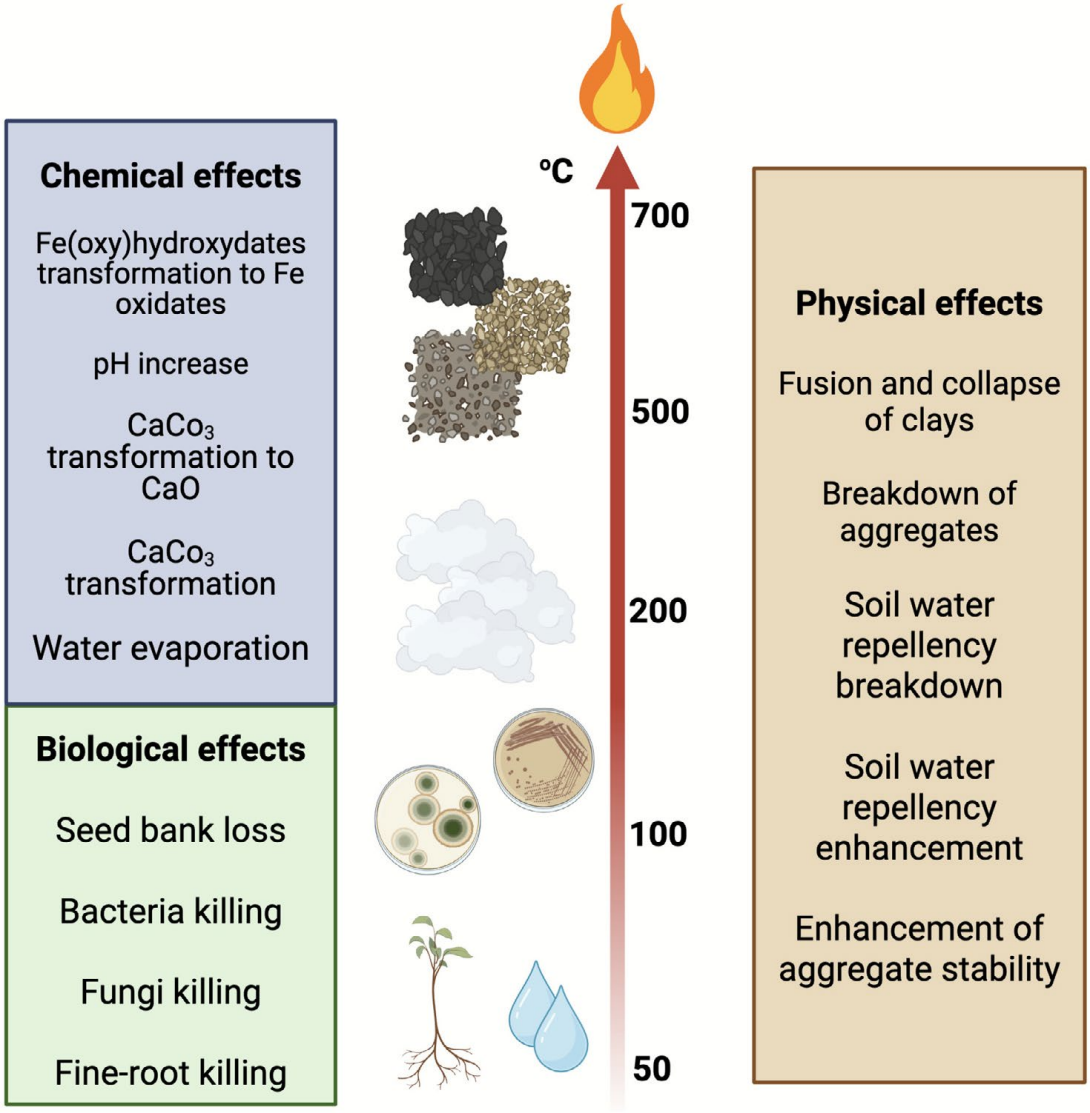
Impact on the biological and physicochemical properties of the soil subjected to extreme temperatures. The indicated temperature ranges are approximate. See text for further details. Figure adapted from Santín and Doerr (2016).

Fire profoundly impacts soil properties, altering its elemental composition and structural characteristics with impacts on microbial diversity and the structure of the communities. These effects vary widely depending on factors such as fire intensity, duration and the pre‐fire condition of the soil (Certini 2005; Agbeshie et al. 2022). The primary impacts of fire on the biological, chemical and physical properties of soil, along with the temperature ranges influencing soil elements and structure, are summarised in Figure 2 based on recent studies. This section provides an overview of the chemical and physical changes induced by fire, followed by a discussion of its biological effects on microbial diversity.

### 3.1 Changes in Soil Chemistry

Fire significantly alters the chemical composition of soil by affecting the availability and transformation of essential nutrients across the temperature ranges reached during combustion (Figure 2). The ash produced by burning can temporarily increase the availability of nutrients such as potassium, calcium and phosphorus, enhancing soil fertility after a fire (Certini 2005). High fire temperatures lead to the combustion of organic matter and minerals, releasing nutrients like nitrogen, sulphur and phosphorus while volatilising elements such as mercury and other trace metals (Roshan and Biswas 2023). Intense heat can also transform soil minerals, forming new compounds and altering the solubility of certain elements, which impacts nutrient storage and cycling (Agbeshie et al. 2022; Roshan and Biswas 2023). Changes in soil physicochemical properties play a critical role in shaping microbial responses. Variations in pH, moisture and organic matter content can favour specific microbial taxa over others (Certini et al. 2005; Certini et al. 2021). For example, underground fires often modify soil pH, moisture levels and organic matter composition, thereby shifting microbial community dynamics. Increased soil acidity from ash deposition can affect nutrient availability, influencing which microbial populations thrive (Barreiro and Díaz‐Raviña 2021).

### 3.2 Changes in Soil Physical Structure

Intense heat can disintegrate soil particles, resulting in a finer soil texture (Figure 2). Under normal temperature conditions, soil texture (defined by the proportions of sand, silt and clay) remains stable and is generally unaffected by fire. However, at elevated surface temperatures, particularly in the mineral soil layer, significant changes can occur. Among these components, clay is the most susceptible to temperature fluctuations. At approximately 400°C, clay hydration and lattice structure integrity begin to degrade (Al‐Khayri and Khan 2024). Even so, research indicates that while clay particles may decrease in favour of silt and sand, the overall soil texture is rarely significantly altered (Flores‐Piña et al. 2024). Intense fires can also induce soil hydrophobicity, making the soil water‐repellent due to the accumulation of burned organic compounds. This hydrophobicity increases runoff and erosion, negatively impacting water infiltration and soil stability (Perei). Additionally, the heat generated by surface fires can alter other soil properties, directly influencing microbial habitats. Changes in soil pH, moisture retention and organic matter content can create conditions that favour certain microbial taxa while disadvantaging others. For instance, increased hydrophobicity can significantly reduce moisture availability, adversely affecting microbial communities (Muñoz‐Rojas and Bárcenas‐Moreno 2019).

### 3.3 Soil Organic Matter

Fire and elevated temperatures alter the composition of soil organic matter, primarily through the thermal decomposition of organic material (Figure 2). This process can lead to reduced soil fertility, changes in soil texture and increased hydrophobicity, all of which negatively impact the soil's water retention capacity (Jiménez‐Morillo et al. 2020). A meta‐analysis by Cheng et al. (2023) revealed that fire‐affected soils show a significant increase in various components of the carbon cycle, including microbial biomass carbon (MBC), dissolved organic carbon (DOC), total carbon (TC), pyrogenic carbon (PyC), soil organic matter (SOM) and soil organic carbon (SOC). However, the effects of fire on soil carbon reservoirs vary widely depending on environmental factors, fire duration, tree species, fire type and soil depth (Agbeshie et al. 2022). Fire exerts complex and multifaceted effects on soil elements and structure. While it can enhance the availability of certain nutrients and induce changes in soil structure, it can also result in organic matter loss, increased hydrophobicity and altered microbial habitats.

## 4 How Do Fires Impact Belowground Microdiversity?

Soil microbial communities respond to fire over the intermediate term, with these responses shaped by both direct factors, such as heat exposure and indirect factors, including changes in vegetation and soil properties (Adkins et al. 2020) (Figure 1). Fire severity plays a crucial role in determining the magnitude and persistence of these changes. Surface fires, in particular, can strongly affect belowground microbiota, altering community composition, diversity and functional roles within the soil ecosystem (Adkins et al. 2020; Barreiro and Díaz‐Raviña 2021). The extent of these impacts depends on fire intensity, which refers to the amount of energy released and the peak temperatures reached; fire duration, indicating how long the soil and organic matter are exposed to elevated temperatures and soil characteristics, such as texture, organic matter content, moisture and structure, which influence heat penetration and microbial resilience. Recent research has highlighted several key effects of surface fires on belowground microbial communities. Surface fires can lead to immediate shifts in microbial community structure. Studies have demonstrated that bacterial and fungal populations often undergo significant changes in their relative abundances after a fire. For instance, certain genera of fire‐resistant bacteria, such as *Firmicutes* and *Actinobacteria*, may increase in abundance, while more sensitive taxa decline (Zhou et al. 2020). These shifts can influence nutrient cycling and organic matter decomposition processes, altering the overall functionality of the soil ecosystem. These changes are influenced by fire severity, frequency and environmental conditions, and they provide insights into how fire can alter microbial communities in soil ecosystems. The following studies present recent findings on how fire affects microbial diversity and functionality, as summarised in Table 1.

**TABLE 1:** Recent works evaluating the modulation of microbial communities in soils affected by surface and underground fires.

Fire type (underground or surface)	Ecosystem affected (e.g., forest, agricultural soil, mine soil)	Impact on microbial abundance and diversity	Seasonal evaluation	Methodology for diversity analysis	References
Surface	*Sierra de la Replana*, Beneixama, Alicante, Spain (warm Mediterranean climate, *Pinus halepensis* vegetation)	Alpha diversity values for the soil bacterial community were higher under moss biocrusts, reaching similar levels to unburned soils, while bare soils had significantly lower bacterial richness and diversity indices. Fire strongly impacted the fungal community, decreasing richness and diversity indices.	February 2020, seven months post‐fire	Amplification of the ITS2 region of fungal rRNA and V4 region of bacterial 16S rRNA	García‐Carmona et al. (2022)
Surface	Cambisol soil (mountain brown soil) covered by *Pinus massoniana* forest	The most abundant phyla in burned soil were *Actinobacteria*, *Firmicutes*, *Proteobacteria*, *Acidobacteria* and *Chloroflexi*.	Fire occurred in July 2009; soil collected in October 2017 from unburned and burned areas	Amplification of the V4–V5 region of the bacterial 16S rRNA gene	Zhang et al. (2021)
Surface	Chaparral stands, California State University San Marcos campus	The only taxon positively affected by hydroseeding was *Firmicutes* in hydroseeded (HYD) areas.	February 2019, five years post‐fire	Amplification of DNA using 16S rRNA primers for the V3–V4 region	Vourlitis et al. (2022)
Surface	Niassa Special Reserve, tropical sub‐humid climate	*Actinobacteria* was negatively affected by fire in sandy and red soils, with higher abundance under low fire frequency (LFF). In oxi‐soils, high fire frequency (HFF) promoted higher abundance of *Actinobacteria*. *Proteobacteria* abundance was significantly affected by fire, while *Acidobacteria* showed no significant differences.	Sites with low (fire return interval > 7 years) and high (fire return interval < 1 year) fire frequency regimes	Amplification of the V3–V4 region of the 16S rRNA gene	Maquia et al. (2021)
Surface	Coniferous and boreal forests	Oligotrophic organisms such as *Actinobacteria* and *Firmicutes* dominated soils three months post‐fire.	August 2015, three months (S1) and 15 years (S2) post‐fire	Amplification of the V4–V5 region of bacterial 16S rRNA genes	Ling et al. (2021)
Surface	Lamto Reserve, Ivory Coast (savanna mosaic with tree density variation and gallery forests)	Total archaeal and bacterial abundances were unaffected by vegetation cover or fire. Fungal abundance was higher under trees and grasses than in bare soil and was not impacted by fire. The bacterial amoA‐AOB gene abundance was higher under bare soil.	January 2017	Real‐Time PCR of total bacteria (16S rRNA), total archaea (16S rRNA), total fungi (18S rRNA) and nitrifying genes (amoA‐AOA and amoA‐AOB)	Srikanthasamy et al. (2021)
Surface	Temperate wet eucalypt forests, Tasmania, Australia	High‐severity fires increased the proportion of *Burkholderiales* and *Firmicutes* while decreasing *Acidobacteriota* and *Alphaproteobacteria*. *Ascomycota* dominated high‐severity soils, while *Basidiomycota* dominated unlogged soils.	40–60 days after regeneration burning	Amplicon sequencing and real‐time quantitative PCR of bacterial 16S rRNA and fungal ITS1 regions	Ammitzboll et al. (2021)
Surface	Permafrost, Subpolar continental climate zone	Abundances of *Acidobacteria* and *Proteobacteria* were unaffected by fire.	September 2011, seven years post‐fire	Amplification of 16S rRNA genes via PCR	Taş et al. (2014)
Surface	Australian Alps, Wellington Plains peatlands	Relative abundance of *Basidiomycota* was lowest in dried peat cores, while *Chloroflexi* abundance was fivefold higher.	March 2015	Amplification of prokaryote (bacteria and archaea) and fungal communities using V4 SSU rRNA primers (515/806) and ITS2 region primers (ITS9/ITS4)	Birnbaum et al. (2023)
Surface	Evergreen shrub bog, Pocosin Lakes National Wildlife Refuge, North Carolina, USA	*Ascomycota* was the dominant fungal phylum, followed by *Basidiomycota*.	Evaluated at 30, 5 and 2 years post‐fire, as well as 71 and 15 days post‐fire	Quantitative PCR (qPCR) of fungal ITS1 region	Tian et al. (2021)
Underground	Coal seams	Community dominated by *Deinococcus–Thermus* (47.7%), *Firmicutes* (34.9%) and *Aquificae* (16.5%).	—	Sequencing and analysis of 16S rRNA gene fragments and PCR amplification	Kadnikov, Mardanov, Beletsky, Grigoriev, et al. (2021)
Underground	Underground coal fire	*Chloroflexi* was the dominant bacterial phylum.	—	PCR amplification of 16S rRNA gene fragments using universal primers 341F and 806R	Kadnikov, Mardanov, Beletsky, Karnachuk, and Ravin (2021)
Underground	Coalmine	*Proteobacteria* and *Acidobacteria* were the most abundant phyla.	April 2013	Amplification of the V1–V2 region of 16S rRNA genes from metagenomic DNA	Banerjee et al. (2020)
Underground	Coal seams	Fire‐enriched members of *Chloroflexi*, *Crenarcheaota* and many unidentified bacterial lineages.	October 2024	Amplification of bacterial and archaeal 16S rRNA V4 region using Illumina MiSeq	Lee et al. (2017)
Underground	Coal fire gas vents	*Firmicutes* was the most diverse group.	August 2008	T‐RFLP analysis and clone libraries of 16S rRNA genes	Zhang et al. (2013)
Underground	Mesotrophic peatland	Abundance of *Alphaproteobacteria* and *Bacteroidetes* cells in burned peat was double that of unaffected areas.	June 2012	Fluorescence in situ hybridisation and analysis of 16S rRNA clone libraries	Belova et al. (2014)
Underground	Trans‐Mexican Volcanic Belt, Mexico	*Firmicutes* increased at higher temperatures, with *Bacillus* as the dominant genus. *Proteobacteria* decreased at higher temperatures. *Ascomycota* abundance increased at 42°C and 50°C compared to control soils.	August 2019	Metagenomic analysis, amplification of V4 regions for archaea and bacteria, and ITS regions for fungi	Flores‐Piña et al. (2024)

Fires can significantly reshape soil microbial communities by altering nutrient availability and soil properties. Certain microbial groups respond rapidly to these changes, with shifts in abundance reflecting both the intensity of the fire and the type of organic inputs released. In this context, Soria et al. (2023) conducted a study on the impact of recent burning on microbial communities, focusing on the phylum *Proteobacteria*. Their findings suggest that the abundance of *Proteobacteria* increased following fire, likely due to the input of organic carbon from low‐severity fires. This study emphasises the role of fire in boosting specific microbial taxa and offers insight into how fire may influence nutrient cycling processes. In a similar context, Sun et al. (2022) found an increase in *Proteobacteria* abundance in soils supplemented with biochar, providing further evidence that fire byproducts and fire‐related soil amendments can alter microbial community composition.

The effects of surface fires on microbial communities are not limited to taxonomic shifts but also extend to functional changes within the soil ecosystem. Certini (2005) highlighted how surface fires can enhance the abundance of microbes involved in nitrogen fixation and organic matter degradation, which in turn can increase nutrient availability in the short term. These changes are critical for understanding the ecological role of microbes in fire‐affected soils.

To further investigate the effects of fire on microbial diversity, Adkins et al. (2020) studied microbial communities in Sierra Nevada mixed‐conifer forests along a burn severity gradient. Their research used both phospholipid fatty acid (PLFA) analysis and 16S rDNA sequencing to assess microbial abundance and diversity. The study found that fungal abundance decreased as fire severity increased, while bacterial diversity was negatively impacted by changes in soil nutrients and texture. This shift was characterised by an increase in *Bacteroidetes* abundance and a decrease in *Acidobacteria*, both influenced by alterations in soil pH. This research contributes to our understanding of how fire‐related changes in soil properties can shape microbial community dynamics.

In a Mediterranean forest context, Lombao et al. (2020) explored the influence of fire regimes (recurrence and severity) on microbial community structure. Using PLFA analysis and community‐level physiological profiling (CLPP), they found that microbial biomass values were negatively correlated with fire severity. Additionally, microbial indices, such as the FungPLFA/BactPLFA and Gram−/Gram+ PLFA ratios, continuously declined with increasing fire severity. These findings underscore how fire frequency and severity can influence microbial functional diversity in Mediterranean ecosystems.

The impact of fire on microbial communities is strongly shaped by long‐term environmental conditions. Yang et al. (2020) examined bacterial communities in a Mediterranean grassland in California, sampling soils nine months after a prescribed burn. Their study, conducted within the long‐term Jasper Ridge Global Change Experiment, found a significant increase in the abundance of Proteobacteria in burned plots. Additionally, they identified 43 keystone taxa, including members of Proteobacteria, Actinobacteria, Bacteroidetes and Firmicutes, highlighting the complex and lasting shifts in bacterial community structure following fire.

In a permafrost context, Zhou et al. (2020) compared bacterial communities in surface and near‐surface permafrost layers across a chronosequence of burned forests in a continuous permafrost zone. They found that functional genes involved in carbon degradation and ammonium oxidation were more abundant in soils recently affected by fire, highlighting the long‐term ecological impact of fire on microbial functions in permafrost ecosystems. However, their study also revealed that changes in soil pH post‐fire were not consistent over time, suggesting that mineral decomposition played a larger role in influencing pH than fire alone.

In another fire‐affected ecosystem, Zhang et al. (2021) examined bacterial communities in fire‐impacted forest soils eight years post‐fire. They found that Actinobacteria, Firmicutes, Proteobacteria, Acidobacteria and Chloroflexi were the most abundant phyla in burned soils. These soils also exhibited higher total carbon and bacterial diversity, leading to increased mineralisation and priming effects. This study highlights the long‐term influence of fire on soil carbon cycling and microbial diversity.

The role of soil cover and vegetation structure can further modulate microbial responses to fire. García‐Carmona et al. (2022) focused on the effects of surface soil fires in Sierra de la Replana, revealing that bacterial alpha diversity was higher in soils with mass biocrusts, similar to unburned soils, while bare soils had significantly lower bacterial richness. This study also noted the strong impact of fire on fungal communities, which saw reduced richness and diversity. These findings underscore the role of biocrusts in mitigating the negative impacts of fire on microbial communities.

Soil type and fire regime can interact to shape microbial dynamics in complex ways. Maquia et al. (2021) investigated the impact of fire on the abundance of *Actinobacteria* and *Proteobacteria* in different soil types. They found that fire negatively affected *Actinobacteria* abundance in sandy and red soils but promoted higher abundance in oxi‐soils with high fire frequency. Additionally, fire frequency significantly influenced *Proteobacteria* abundance, providing further evidence that soil type and fire regime can interact to shape microbial communities.

To understand the broader effects of fire on microbial communities in California chaparral ecosystems, Vourlitis et al. (2022) studied surface soils from unburned, burned and hydroseeded chaparral stands. Their findings revealed that hydroseeding increased the abundance of *Firmicutes* in burned soils, while copiotrophic taxa like *Proteobacteria*, *Bacteroidetes*, *Actinobacteria* and *Firmicutes* dominated the bacterial community. This highlights the potential for post‐fire restoration efforts to influence microbial community composition.

In a wet savanna ecosystem, Srikanthasamy et al. (2021) investigated the short‐term effects of fire on microbial and nitrifier communities, focusing on how vegetation cover mediates these responses. Wet savannas are characterised by a seasonal rainfall regime and a heterogeneous vegetation structure, which can create microhabitats that buffer soil microbial communities from environmental stressors, including fire. Their study found that bacterial and archaeal abundances were largely unaffected by fire or vegetation cover, suggesting resilience of these groups to short‐term fire disturbances. In contrast, fungal abundance was significantly higher under trees and grasses compared to bare soils, indicating that vegetation provides favourable conditions for fungal colonisation and activity after fire. These findings underscore the crucial role of vegetation in shaping post‐fire microbial dynamics and highlight how plant–soil–microbe interactions can influence ecosystem recovery following fire events.

The diversity of microbial communities in forest soils affected by varying fire severities was documented by Ammitzboll et al. (2021), who found that high‐severity burns increased the abundance of *Burkholderiales* and *Firmicutes*, while low‐severity burns favoured *Acidobacteria* and *Alphaproteobacteria*. Fungal communities were dominated by *Ascomycota* in high‐severity burns and *Basidiomycota* in unlogged soils, illustrating how fire severity can shape both bacterial and fungal community composition.

In subtropical peatlands, Tian et al. (2021) investigated fungal community responses to fire. They found that *Ascomycota* was the most abundant phylum, followed by *Basidiomycota*. The dominance of *Archaeorhizomycetes*, a class within *Ascomycota*, was associated with root symbionts and slow‐growing species, suggesting its resilience to fire‐related disturbances.

Taş et al. (2014) explored the impact of fire on microbial communities in Alaskan boreal forest soils, finding that the abundance of *Acidobacteria* and *Actinobacteria* was unaffected by fire. However, enzymatic activities related to cellulose and hemicellulose degradation increased in burned areas, suggesting that fire may promote specific microbial functions, despite minimal changes in community composition.

In Australian bog ecosystems, Birnbaum et al. (2023) compared microbial communities in degraded and intact peatlands with histories of grazing and fire. They reported that Chloroflexi abundance was significantly higher in dried peat cores, while Basidiomycota abundance was lower, highlighting how fire can selectively alter microbial community structure in peatland soils. Collectively, these studies demonstrate that the effects of fire on microbial communities are context‐dependent, influencing both composition and functional dynamics across different ecosystems.

## 5 Effect of Underground Fires on Microbial Soil Diversity

Underground fires, which occur when organic materials below the soil surface ignite and smoulder, can have profound effects on soil microbiota. Unlike surface fires, underground fires often maintain high soil temperatures—ranging from 50°C to 200°C depending on fuel type and depth—and can persist for extended periods, from several days to weeks or even months (Torero et al. 2020). This prolonged exposure, combined with limited oxygen availability, creates a unique thermal and chemical environment that can selectively alter microbial community composition, reduce diversity and shift functional roles within the soil ecosystem.

Studies have shown that certain groups, particularly thermophilic and anaerobic bacteria, may thrive in the aftermath of underground fires due to their ability to withstand elevated temperatures (Flores‐Piña et al. 2024). For example, taxa from the phylum *Firmicutes* increase in abundance, while more sensitive groups, such as *Proteobacteria*, may decline (Flores‐Piña et al. 2024). The following describes some of the most recent studies on underground fires and their effects on microbial communities, as summarised in Table 1.

Zhang et al. (2013) examined the bacterial diversity of coal fire gas vents using Terminal Restriction Fragment Length Polymorphism (T‐RFLP). Their findings revealed that *Firmicutes* was the most diverse and dominant group in all the samples, followed by *Proteobacteria*, which was the second most abundant group. Similarly, Belova et al. (2014) used fluorescence in situ hybridisation and analysis of 16S rRNA gene clone libraries to study bacterial community composition in the Galitskii Mokh mesotrophic peatland and examine structural changes following the 2010 wildfire. They demonstrated that the abundance of *Alphaproteobacteria* and *Bacteroidetes* cells in the peat of the burned site was twice as high as in the unaffected area.

Extreme environmental conditions, such as intense heat, soil disturbance and prolonged exposure to combustion, can strongly influence microbial community structure and function. Both Lee et al. (2017) and Banerjee et al. (2020) investigated bacterial communities in environments affected by extreme conditions, though their study sites varied significantly in nature. While Lee et al. (2017) focused on the diversity of bacterial and archaeal communities in soils impacted by active fire fronts in Centralia, Banerjee et al. (2020) examined bacterial communities in the Bera coal mine in India, an area subjected to continuous excavation and coal burning. Despite these differences in environmental conditions, both studies revealed distinct shifts in microbial community composition linked to extreme heat and disturbance. Lee et al. (2017) observed that fire‐affected soils were enriched with members of *Chloroflexi*, *Crenarchaeota* and numerous unidentified bacteria, with the hottest soils exhibiting the most variable communities. Similarly, Banerjee et al. (2020) found that the bacterial communities in the coal mine were dominated by *Proteobacteria* and *Acidobacteria*, reflecting a high degree of adaptability to harsh, fire‐affected environments. Both studies underscore the resilience of microbial communities in extreme environments and the distinct microbial groups that thrive in the aftermath of fire and other disturbances.

Underground coal seam and pit fires create extreme thermal and chemical conditions that profoundly affect soil microbial communities. Kadnikov, Mardanov, Beletsky, Grigoriev, et al. (2021) analysed 16S rRNA gene fragments from soils affected by underground coal seam burning using PCR amplification and metagenomic sequencing, revealing a community dominated by Deinococcus–Thermus (47.7%), Firmicutes (34.9%) and Aquificae (16.5%). In a related study, Kadnikov, Mardanov, Beletsky, Karnachuk, and Ravin (2021) investigated soils from an abandoned open coal pit near Kiselevsk, Kemerovo Region, finding that Chloroflexi, particularly the class Ktedonobacteria—commonly associated with natural CO2 gas vents—dominated the microbial community. Both studies employed PCR amplification of 16S rRNA gene fragments with universal primers 341F and 806R to characterise bacterial diversity. More recently, Flores‐Piña et al. (2024) examined microbial communities along temperature gradients in soils impacted by an underground fire in the Trans‐Mexican Volcanic Belt. Their results showed that Firmicutes increased in abundance with rising temperatures, while Proteobacteria decreased relative to unaffected soils. Fungal diversity was also strongly affected, and while spore formation is considered a key survival mechanism, the authors suggested that thermotolerance may additionally promote resilience in these communities. Table 1 summarises selected studies on surface and underground fires, highlighting how these events modulate soil microbial diversity and function.

## 6 How Do Soil Microbes Respond to Heat?

Undoubtedly, fires impact soil microbial communities, which play a crucial role in soil health. While some microbial populations may be killed during a fire, others can survive or rapidly recolonise the area. This shift can alter nutrient cycling processes and affect soil health post‐fire (Bouskill et al. 2022). Post‐fire environments present unique challenges and opportunities for soil microbial communities. The effects of fire on soil microbial responses can vary depending on the intensity and type of fire, as well as the pre‐existing soil conditions. Recent studies highlight several key responses of soil microbes following fire events (Barreiro and Díaz‐Raviña 2021).

Soil microbial communities respond to fire in the intermediate term, with these responses shaped by both direct factors, such as heat exposure, and indirect factors, such as changes in vegetation and soil properties (Certini et al. 2005; Adkins et al. 2020). Direct effects of fires on soil microbiota include the destruction of litter and humus, resulting in the loss of habitat and microbial death due to heat, which often leads to a reduction in microbial biomass. Surface soil temperatures during wildfires can range from 200°C to 700°C, while temperatures in deeper layers typically remain between 40°C and 150°C, depending on fire intensity, duration and soil moisture (Köster et al. 2021).

The resilience of soil microbial communities after fire is critical for ecosystem recovery. Studies show that while some microbial groups can quickly reestablish, others may take longer to recover. For instance, the recovery of mycorrhizal fungi is often slower compared to bacteria, which can hinder plant regrowth and overall soil health (Hopkins et al. 2024). Understanding these dynamics is essential for effective land management practices in fire‐affected areas.

Following a surface fire, microbial communities can exhibit resilience and recovery, often re‐establishing their pre‐fire composition within months to years, depending on environmental conditions and fire severity. Studies have shown that certain microbial taxa can recolonise rapidly, while others may take longer to recover, indicating varying levels of resilience within the community (Pulido‐Chavez et al. 2023). In a study of 20‐year fire chrono sequences elaborated by (Perez‐Valera et al. 2020), it was documented that some taxa, such as *Ascomycota* and *Firmicutes*, were favoured soon after fire and identified as the most resilient. Likewise, the dominance of *Firmicutes* was attributed to spore formation.

On the other hand, the resilience and recovery of soil microbiota after underground fires are critical for ecosystem recovery. Some studies suggest that microbial communities can take longer to recover compared to those affected by surface fires due to the more severe thermal impacts (Barreiro and Díaz‐Raviña 2021). However, certain resilient taxa can recolonise quickly, facilitating recovery and stabilising the soil ecosystem over time.

In a study conducted by Chamizo et al. (2020), *Phormidium ambiguum* and *Scytonema javanicum* (Cyanobacteria) were inoculated into burned soils, resulting in a significant increase in chlorophyll a content, reduced hydrophobicity and enhanced soil penetration resistance, particularly in soils with finer texture and higher organic content after 45 days of inoculation.

Likewise, studies have shown that fire can lead to an initial decline in microbial diversity, followed by a shift as communities recover. For instance, *Bacteroidetes* and *Actinobacteria* frequently increase in abundance post‐fire, while sensitive groups such as some *Proteobacteria* may decrease (Jiang et al. 2025). This shift can influence ecosystem functions, including nutrient cycling and organic matter decomposition.

Fire impacts not only the composition but also the functional capabilities of soil microbial communities. Research indicates that microbial metabolic activities, such as carbon and nitrogen cycling, can be enhanced in the immediate aftermath of a fire. For example, enhanced enzyme activity related to the degradation of organic materials has been observed, suggesting increased decomposition rates (Pei et al. 2023). However, long‐term functional recovery can be variable, often depending on subsequent environmental conditions. This shift can lead to short‐term increases in nutrient availability, which may benefit plant regrowth in the affected areas.

### 6.1 Molecular Responses

Microorganisms have developed different strategies to survive under adverse conditions such as extreme heat (Li and Gänzle 2016; Setlow 2006). Of course, archaea and certain bacterial groups like Firmicutes are experts in this field, and some of their species are well known for their thermotolerance or thermophily (Straub et al. 2018). But how do they manage to survive the ravages of fire and the subsequent effects, such as sustained high temperatures? Upon exploring the molecular mechanisms involved, one of the main strategies is spore production and DNA protection and repair, with species of the genus *Bacillus* being extensively studied. Spores are resistance structures that allow microorganisms to remain dormant until conditions improve, enabling them to germinate and reproduce again. Spores exhibit greater heat resistance, particularly to ‘dry heat,’ although ‘wet heat’ can also induce their formation. An interesting fact is that spores from thermophilic organisms, such as archaea, are not more heat‐resistant than those from mesophiles, like *Bacillus*. In fact, the intrinsic resistance of spores to dry heat also implies resistance to desiccation (Setlow and Johnson 2012). For example, spores of *Bacillus subtilis* are resistant to multiple cycles of drying and rehydration. According to Setlow (2006), one of the main internal structures damaged by heat is DNA, so having efficient repair and recombination systems is crucial for survival. Genes such as *recA* in *B. subtilis* are essential for heat stress resistance (among others like radiation), whether or not spores are formed. Other genetic elements and their protein products, such as *exoA*, *endoIV* (*yqfS*) and *ykoUVW*, are transcribed in the developing spore. These enzymes are involved in repairing DNA damage caused by dry heat in wild‐type spores, where the activation of several DNA repair system genes is coordinated by σ^G^, playing a fundamental role in resistance to dry heat stress. Experiments in *Escherichia coli* have shown that mutants in genes such as σ^H^ and σ^S^, as well as strains deficient in the production of proteins SodA/B, IbpA/B, and in colanic acid synthesis, are more sensitive to heat stress compared to their isogenic parental wild‐type strains (Li and Gänzle 2016).

During fire exposure, cells undergo protein denaturation or unfolding (Katikaridis et al. 2021). Therefore, molecular chaperones, including the heat shock proteins (Hsps), are a general feature of microbial cells, where these proteins help cope with heat. One of the most studied heat shock proteins is Hsp90, as it is ubiquitous not only in microbial cells but also present in human cells; it plays fundamental roles in proteostasis, facilitating folding, disaggregation, prevention of aggregation, degradation, activation and protection against degradation of various cellular proteins. According to Wickramaratne et al. (2024), Hsp90 is a good team player in bacteria, as it works together with the Hsp70 molecular chaperone and Hsp70 cochaperones to remodel proteins.

**FIGURE 3:**
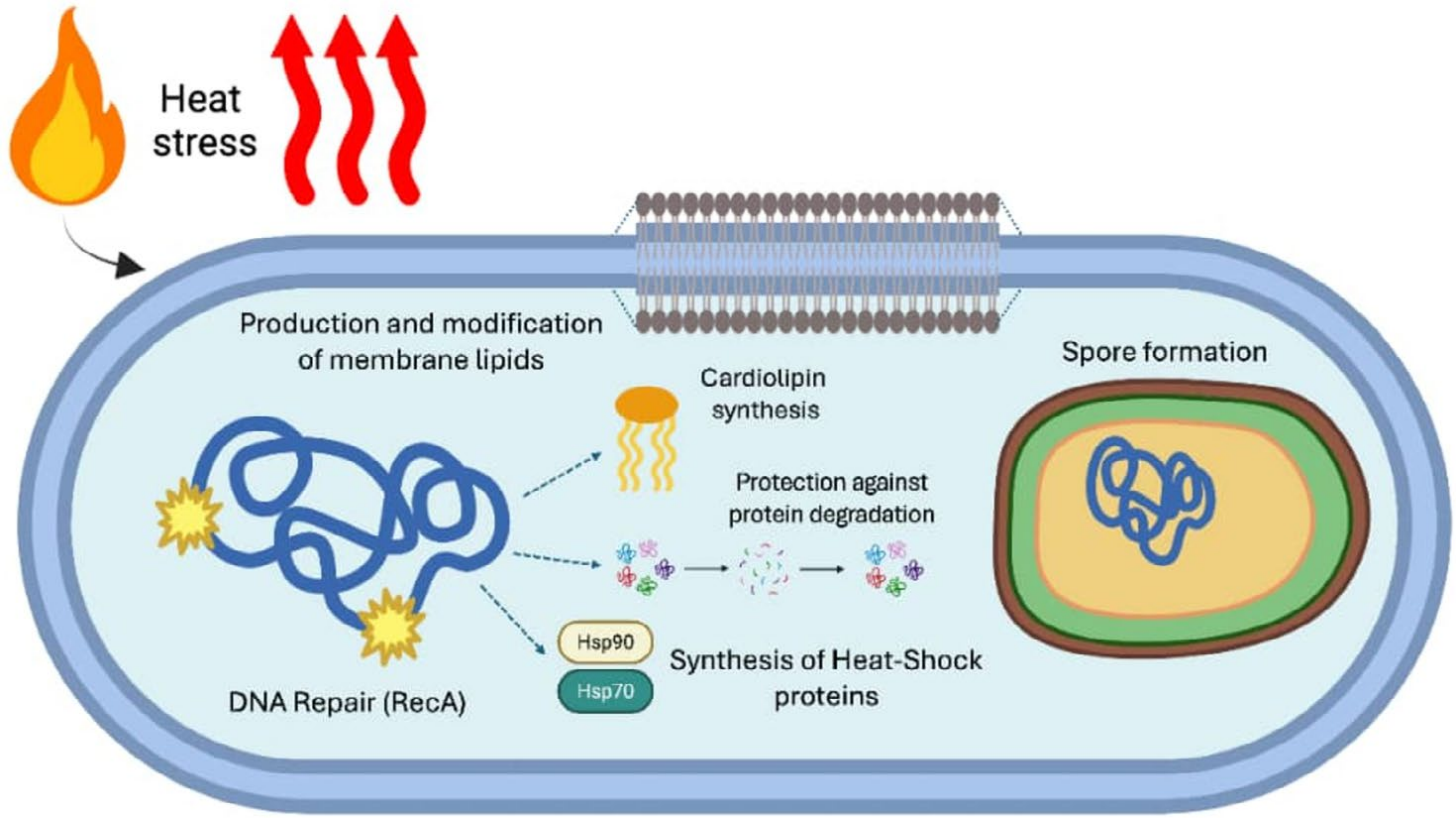
General mechanisms employed by bacterial cells to survive heat stress. Key adaptive strategies used by bacteria (e.g., *Bacillus* spp.) to withstand elevated temperatures include spore formation, DNA protection and repair, protein folding by heat shock proteins (Hsps) and modifications in membrane lipid composition. DNA repair genes such as *recA* play a crucial role in repairing heat‐induced DNA damage. Heat shock proteins, including Hsp90 and Hsp70, assist in protein stabilisation and refolding. Additionally, bacteria adjust membrane lipid production (e.g., cardiolipin) and composition to preserve membrane integrity and function under stress.

Another molecular mechanism involved in the heat response in bacteria is the modification of membrane components. Bacterial cells respond to alterations in the membrane structure through various molecular mechanisms to synthesise new lipids and modify existing membrane lipids in order to achieve lipid homeostasis (Shelake et al. 2024). The main classes of lipids include glycerophospholipids, glycerolipids and prenol lipids. It has been observed that the bacterial membrane exhibits a mixture of negatively charged cardiolipin (CL), phosphatidylglycerol (PG) and zwitterionic phosphatidylethanolamine (PE). PEs are the main phospholipids in Gram‐negative bacteria, whereas in Gram‐positive Enterobacteriaceae and bacilli, PE is not found. In the same review, it is proposed that the isomerisation of unsaturated fatty acids from the *cis* to *trans* configuration plays an important role as an adaptive response to heat stress (as well as other types of stress such as heavy metals, organic solvents or antibiotics), as increasing the lipid packing order in the membrane reduces its permeability (Lee et al. 2017). Figure 3 illustrates the above general mechanisms of bacterial cells to survive heat stress.

## 7 Conclusions and Perspectives

Despite the initial adverse effects, many soils can recover over time. Vegetation regeneration helps restore soil properties, although this may take many years, depending on the intensity of the fire and environmental conditions (Cheng et al. 2023). The magnitude of the effects of a forest fire on the soil depends on several factors, such as fire intensity, the vegetation that burns, soil type, the duration of high temperatures and the climatic conditions following the fire (Agbeshie et al. 2022). In summary, fires have complex and lasting effects on soil properties, which can impact ecosystem regeneration and soil quality. However, recovery is possible with proper measures and over time, depending on the severity of the fire and restoration efforts.

Soil microbial responses to post‐fire impacts are complex and multifaceted, involving shifts in community composition, changes in functional capabilities and alterations in soil physicochemical properties. These responses play a crucial role in ecosystem recovery and highlight the need for ongoing research to inform restoration strategies and crop productivity. Understanding these dynamics is critical for developing effective land management and restoration strategies in fire‐affected areas. As ecosystems recover, monitoring microbial responses can provide valuable insights into the resilience and health of soil systems. Ultimately, both surface and underground fires present complex challenges and opportunities for soil microbiota, shaping the ecological landscape in significant ways.

## Author Contributions

**Ma. del Carmen Orozco‐Mosqueda:** writing – original draft, writing – review and editing. **Aurora Flores‐Piña:** writing – original draft, writing – review and editing. **Ajay Kumar:** writing – original draft, writing – review and editing. **Fannie I. Parra‐Cota:** writing – original draft, writing – review and editing. **Sergio de los Santos‐Villalobos:** writing – original draft, writing – review and editing. **Debasis Mitra:** writing – original draft, writing – review and editing. **Olubukola Oluranti Babalola:** conceptualisation, writing – original draft, writing – review and editing. **Gustavo Santoyo:** conceptualisation, writing – original draft, writing – review and editing.

## Conflicts of Interest

The authors declare no conflicts of interest.

## Data Availability

Data sharing not applicable to this article as no datasets were generated or analysed during the current study.
